# Overall survival of individuals with metastatic cancer in Sweden: a nationwide study

**DOI:** 10.1186/s12889-022-14255-w

**Published:** 2022-10-14

**Authors:** Greta Bütepage, Peter Carlqvist, Johanna Jacob, Asbjørn Toft Hornemann, Simona Vertuani

**Affiliations:** 1 Nordic Market Access AB, 113 59 Stockholm, Sweden; 2 Novartis Sverige AB, 164 28 Kista, Sweden; 3 Novartis Norge AS, 0401 Nydalen, Norway

**Keywords:** Metastatic cancer, Overall survival, Long-term survival, Retrospective registry study

## Abstract

**Aims:**

Consistent improvements for overall survival (OS) have been reported for individuals with metastatic cancer. Swedish population-based registers allow national coverage and long follow-up time. The aim of this study was to estimate and explore long-term OS of individuals diagnosed with metastatic cancer using Swedish nationwide health registers.

**Methods:**

Individuals with metastatic breast (MBC), non-small cell lung (MNSCLC), ovary (MOC) or colorectal cancer (MCRC) or metastatic malignant melanoma (MMM) were identified in the Swedish national cancer register and national patient registers. Survival was estimated and stratified by available variables. Potential cure fractions were estimated using mixture cure models.

**Results:**

In total, approximately 69,000 individuals were identified. The most common cancers were MCRC (36.2%) and MNSCLC (29.5%). Men were more frequently diagnosed with MNSCLC, MCRC, and MMM compared to women. Except for MOC, about 50% of individuals were 70 years or older at diagnosis. Throughout the study period survival differed across cancers. The longest median OS was observed for individuals with MOC and MBC. At 10 years of follow-up, the survival curves flatten at a survival rate of approximately 10% for all cancers except MNSCLC. The youngest age groups had the longest median OS. Increased survival was also observed for individuals diagnosed in 2015 and 2018 compared to individuals diagnosed during earlier years. The estimated cure fractions were 4% for MBC, 1.5% for MNSCLC, 6.8% for MCRC, 8.6% for MOC and MMM.

**Conclusions:**

Long-term survival has been assessed across all indications except for NSCLC.. The findings may be relevant for healthcare planning to meet the needs of future patients and potential long-term survivors.

**Supplementary Information:**

The online version contains supplementary material available at 10.1186/s12889-022-14255-w.

## Introduction

Most cancers in the metastatic stage are associated with poor prognosis and high mortality rates. Studies in high-income countries have shown consistent improvements in cancer survival over time [1, 2]. Reasons for the observed improvement in cancer survival are likely to be multifactorial including healthcare reforms and technological advances. These may result in earlier and more precise diagnosis, more effective and targeted treatment, and optimized patient management. With the continuously improving overall survival (OS), a proportion of individuals diagnosed with advanced malignancies may experience an OS close to that of the general population. However, advancements of OS differ across indications [1, 2].

Increasing long-term survival rates raise new clinical questions and challenges. With metastatic cancer becoming chronic, long-term survivors may face complex issues regarding their disease and treatment. In combination with disease management, increasing cancer survivorship may also result in a substantial economic burden for public health systems [3]. Although the economic burden is greatest shortly after diagnosis, it remains high throughout the remaining years of life [3]. This is relevant for public health planning and resource allocation on a societal level. Informed decision making within public health systems requires detailed knowledge on cancer survival to meet future health care demands and challenges.

Monitoring trends in patient survival facilitates the assessment of the treatment advancements within oncology [4]. OS has traditionally been accepted as the gold standard among oncology efficacy endpoints. The majority of research describes OS in clinical trials, but follow-up times often do not allow for reporting survival rates beyond five years [5]. There is a lack of long-term survival data for metastatic cancer in a real-world nation-wide setting.

In Sweden, government-administered health registries allow researchers to follow patients throughout the entirety of their life and obtain information on e.g., diagnoses, prescribed medication, and death [6]. These population-based registers allow for large sample sizes with close to complete coverage and long follow-up times. This facilitates comprehensive survival estimates and valuable insights into long-term survival within oncology in a real-world setting.

The aim of this study was to estimate and explore long-term OS of individuals diagnosed with metastatic cancer with the aid of Swedish nationwide health registers. The indications of interest were selected based on the high incidence and occurrence of metastases and included metastatic breast cancer (MBC), metastatic non-small cell lung cancer (MNSCLC), metastatic colorectal cancer (MCRC), metastatic ovarian cancer (MOC) and metastatic malignant melanoma (MMM). Estimating the OS of these advanced malignancies in Sweden could be beneficial for resource allocation and healthcare planning, to meet the needs of future patients and potential long-term survivors.

## Methods

### Data

This study was a retrospective, observational cohort study using Swedish nationwide, population-based, administrative health registries covering the entire specialist care in Sweden. Individuals with metastatic disease were of interest including de novo metastatic cancer, i.e., metastatic cancer at primary diagnosis, and recurrent metastatic cancer, i.e., metastatic cancer at disease recurrence. Individuals with a de novo diagnosis were identified in the Swedish Cancer Register (SCR) using International Classification of Diseases – 10^th^ revision (ICD-10) codes (MBC: C50, MNSCLC: C34, MCRC: C18 – C21, MOC: C56, MMM: C43). Only individuals with a code indicating the presence of metastases (M1) at diagnosis according to the globally recognized TNM Classification of Malignant Tumours were included.

The SCR does not routinely provide data on disease recurrence. Consequently, this administrative dataset is limited due to a lack of essential prognostic information. The identification of the relevant patient population is challenging. To identify individuals with a disease recurrence, the Swedish National Patient Register (SNPR) and the SCR were linked using the unique personal identity numbers (PIN) issued to all residents in Sweden [7]. Metastatic tumour recurrence was defined as the presence of ICD-10 codes C78 or C79 in the SNPR if a relevant primary cancer diagnosis was registered in the SCR prior to the first instance of a secondary malignancy code. It was assumed that any occurrence of C78 or C79 would be linked to the preceding cancer diagnosis. Individuals diagnosed between January 2005 and December 2018 were included in the study. Additional information was extracted on age at diagnosis, year of diagnosis, sex, and type of diagnosis.

### Analysis

Descriptive statistics were applied to summarize patient numbers, and characteristics. To simplify data and data privacy concerns, age at diagnosis was categorized into five groups: < 50, 50–59, 60–69, 70–79 and 80 + years. Similarly, year of diagnosis was categorized into three groups: 2005–2009, 2010–2014, 2015–2018. The type of diagnosis distinguished between de novo and recurrent disease.

Survival time was calculated from date of diagnosis until date of death. Individuals were censored in case of emigration, or if they were alive at the end of follow-up. Long-term survival was defined as survival at ≥ 10 years after diagnosis of metastatic disease. The observed median OS, expressed in months including 95% confidence intervals (Cis), was estimated using Kaplan–Meier method [4]. Additionally, one-, five and 10-year survival rates were estimated for all indications. These times are commonly used as a summary measure to describe the survival of a group of individuals [4].

To assess the effect of available patient characteristics, survival was stratified. All available variables, i.e., sex, age at diagnosis, year of diagnosis and type of diagnosis, resulted in distinct survival patterns across indications when assessing the Kaplan–Meier curves. A multiple Cox regression was performed for all indications to assess all variables in a regression model. Both crude and adjusted hazard ratios were reported. The assumption of proportionality was assessed graphically using Schoenfeld residuals and log cumulative hazard plots. Considering the long follow-up and potential violation of the proportional hazard assumption the results of the regression should be interpreted with caution.

At present, there are no diagnostic nor statistical tests that can assess whether an individual is cured of cancer. Instead, long-term follow up represents the only way to approximate cure rates, i.e., long-term survival. For studies with limited follow-up and a possibly heterogeneous patient population, cure models may provide preliminary long-term survival estimates and probabilities of cure [8]. In the present study six standard parametric mixture cure models were applied to estimate the cure fraction. Standard cure fraction models assume that the study population includes both susceptible individuals, who experience the event of interest and non-susceptible individuals that will not [9]. This allows estimation of the proportion of long-term survivors as well as the proper survival function of susceptible individuals. The model selection was based on goodness of fit statistics, namely Akaike information criterion and Bayesian information criterion. Additionally, the model fit was assessed visually to ensure clinical validity. The best fitting models including the estimated cure fractions are presented for each indication.

## Results

**Fig. 1:**
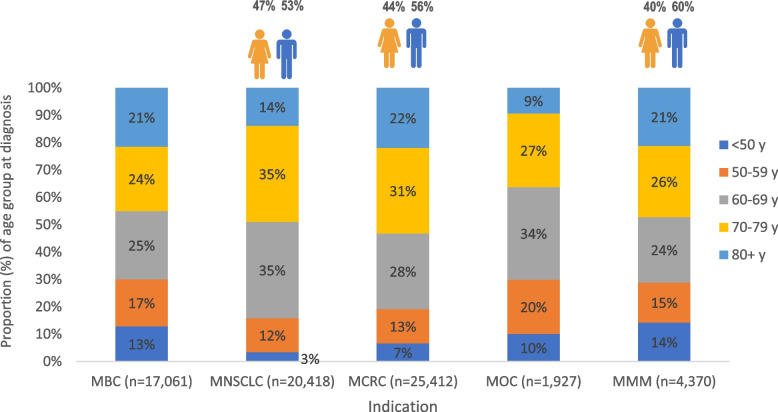
Overview of age and sex for all indications. MBC: Metastatic breast cancer, MNSCLC: Metastatic non-small cell lung cancer, MCRC: Metastatic colorectal cancer, MOC: Metastatic ovarian cancer, MMM: Metastatic malignant melanoma, y: years

The indication with the largest number of individuals identified was MCRC followed by MNSCLC, MBC, MMM and MOC (Table 1). Most individuals were included based on a secondary, recurrent diagnosis (> 50%). Only for MNSCLC, a de novo disease represented the majority (76%) of included individuals. For MOC, no information on type of diagnosis was made available due to data privacy reasons (low patient numbers). Figure 1 shows the age distribution across the indications. On average, individuals with MOC were the youngest (Mean age: 64.5 years) compared to the other indications (MBC: 67.3 years, MNSCLC: 68.9 years, MCRC: 69.4 years, MMM: 66.5 years). More men than women with MNSCLC, MCRC or MMM (Fig. 1).

**Table 1:** Number of individuals, by indication and time of diagnosis

Indication	Type of diagnosis	Number of individuals (n)			Total
		2005—2009	2010—2014	2015—2018	
**Metastatic breast cancer**	De novo	616	772	694	17,061
	Recurrent	5,447	5,346	4,186	
**Metastatic lung cancer**	De novo	4,738	5,905	4,799	20,418
	Recurrent	1,785	1,811	1,380	
**Metastatic colorectal cancer**	De novo	3,981	4,569	3,851	25,412
	Recurrent	4,587	4,578	3,846	
**Metastatic ovarian cancer**	-	584	755	588	1,927
**Metastatic malignant melanoma**	De novo	90	80	65	4,370
	Recurrent	1,253	1,494	1,388

Throughout the study period survival differed across all indications (Table 2 and Fig. 1a–e supplementary material). The longest median survival was observed for individuals with MOC and MBC (23.4 and 20 months, respectively). The largest 5-year survival of > 20% was observed for MBC and MOC followed by MCRC and MNSCLC. At 10 years of follow-up, however, the survival curves flatten at approximately 10% for all indications except MNSCLC. The 10-year survival for MNSCLC was estimated to 1.7%.

**Table 2:** Landmark survival for all indications

Survival	Metastatic breast cancer	Metastatic non-small cell lung cancer	Metastatic colorectal cancer	Metastatic ovarian cancer	Metastatic malignant melanoma
Median survival (months, 95% CI)	20 (19.4, 20.6)	5.18 (5.1, 5.3)	12.8 (12.6, 13.1)	23.4 (22.0, 24.9)	7.4 (6.8, 7.9)
1-year survival (%, 95% CI)	61 (60.4, 61.9)	25.4 (24.8, 26.0)	51.8 (51.1, 52.4)	69 (66.9,71.1)	38.9 (37.4, 40.4)
5-year survival (%, 95% CI)	21.6 (20.9, 22.3)	3.2 (3.0, 3.6)	15.1 (15.0, 16.0)	20.3 (18.3, 22.5)	15.6 (14.4, 16.9)
10-year survival (%, 95% CI)	10 (9.4, 10.7)	1.7 (1.4, 2.0)	10 (9.5, 10.5)	10.3 (8.54, 12.5)	10.6 (9.4, 12.1)

**Fig. 2:**
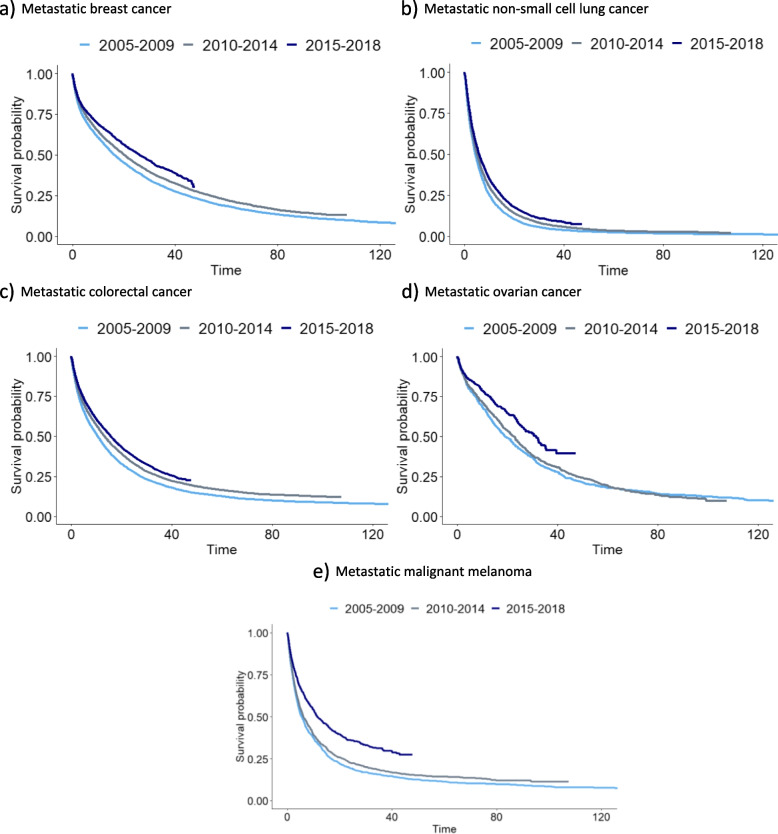
Overall survival for all indications, by year of diagnosis. **a** Metastatic breast cancer, **b** Metastatic non-small cell lung cancer, **c** Metastatic colorectal cancer, **d** Metastatic ovarian cancer, **e** Metastatic malignant melanoma

OS was further stratified by sex, type and year of diagnosis, and age at diagnosis (Table 3).

**Table 3:** Observed median survival, by indication, sex, type of diagnosis, age at diagnosis, and year of diagnosis

Patient characteristics		Median survival (months, 95% CI)				
		**Metastatic breast cancer**	**Metastatic non-small cell lung cancer**	**Metastatic colorectal cancer**	**Metastatic ovarian cancer**	**Metastatic malignant melanoma**
**Type of diagnosis**	De novo	20.8 (19.6, 22.8)	5.5 (5.3, 5.6)	13.8 (13.3, 14.2)	NA	12.7 (9.9, 15.3)
	Recurrence	19.8 (19.3, 20.5)	4.16 (4, 4.4)	11.7 (11.3, 12.2)	NA	7 (6.5, 7.6)
**Age at diagnosis**	< 50 y	30.9 (29.1, 33.0)	8.7 (7.8, 10.7)	21.5 (19.9, 23.2)	33.3 (26.2, 40.7)	11.6 (10.2, 12.9)
	50 – 59 y	27.6 (25.9, 29.5)	6.43 (6.2, 6.9)	22.5 (21.1, 24)	29.2 (25.8, 35.1)	11.2 (9.7, 13.1)
	60 – 69 y	23.9 (22.3, 25.3)	5.8 (5.6, 6.0)	18.4 (17.6, 19.1)	24.1 (21.4, 27.1)	9.1 (7.8, 10.4)
	70 – 79 y	18.8 (17.5, 19.9)	4.59 (4.4, 4.8)	11.7 (11.2, 12.2)	21.2 (18.3, 24.1)	6.2 (5.2, 7.3)
	80 + y	7.9 (7.3, 8.6)	3.5 (3.3, 3.8)	5 (4.7, 5.3)	11.6 (7.8, 14.7)	3.2 (2.9, 3.8)
**Year of diagnosis**	2005 – 2009	16.4 (15.4, 17.4)	4.5 (4.3, 4.7)	10.5 (10.2, 11.0)	19.2 (17.1, 22.4)	5.4 (4.8, 6.3)
	2010 – 2014	20.3 (19.4, 21.2)	5.2 (4.9, 5.4)	13.3 (12.8, 13.8)	22.6 (20.1, 24.7)	6.1 (5.6, 7.2)
	2015 – 2018	26.2 (24.8, 28.2)	6 (5.7, 6.3)	15.7 (15.0, 16.5)	31.5 (26.5, 35.4)	11.5 (10.5, 13.5)
**Sex**	Men	NA	4.8 (4.6, 4.9)	13.8 (13.3, 14.2)	NA	6.8 (6.2, 7.6)
	Women	NA	5.7 (5.5, 5.9)	11.7 (11.3, 12.2)	NA	8.2 (7.3, 9.6)

*CI* Confidence interval, *y* year

The youngest age groups (< 50 and 50–59 years) had the longest median survival regardless of indication. Increased survival was also observed for individuals diagnosed in 2015 and 2018 compared to individuals diagnosed during earlier years (Fig. 2a–e).

The Cox regression showed that age at diagnosis and the year of diagnosis had statistically significant impact on survival for all indications (Tables 4, 5, 6, 7 and 8 – supplementary material). For all indications, an age of > 60 years at diagnosis was linked to a significantly increased hazard of death. Individuals with MOC were on average the youngest (64.5 years) and comprised the fewest individuals aged 70 years or older across the five indications. The longest median survival was observed for this group. The hazard ratio (HR) for individuals diagnosed between 2015 and 2018 was approximately 0.7 compared to individuals who were diagnosed 2005–2009 regardless of indication.

The type of diagnosis had a significant effect on the hazard of death comparing de novo disease (reference group) with recurrent disease for individuals with MNSCLC or MCRC (Tables 5 and Table 6). Sex had a significant effect on survival for individuals with MNSCLC or MMM (Tables 5 and Table 8). The fit of the mixture cure models was assessed visually and based on goodness-of-fit statistics (Table 9—supplementary material). The generalized gamma model was the best fitting model for all indications except for MBC. The estimated cure fractions ranged between 1.5% (MNSCLC) and 8.6% (MOC and MMM).

## Discussion

### Observed overall survival estimates and long-term survival

For all cancers, the survival curves declined rapidly at the beginning of follow-up. The increased mortality during the initial years indicates challenges regarding disease control and prevention of further metastases. Towards the end of follow-up, however, the survival plateaued. Individuals alive at that time had a relatively low mortality (conditional survival).

The proportion of individuals alive at 10 years was the lowest for MNSCLC (< 2%). This was expected as lung cancer continues to be linked to a poor prognosis and high fatality rate [10].

The limited data on long-term survival corroborates the poor prognosis linked to MNSCLC. The observed OS for MNSCLC in the present study is in line with previously reported survival estimates. Studies utilizing data from the Surveillance, Epidemiology, and End Results (SEER) registry report a median survival of 4 months for individuals with MNSCLC [11, 12], however, higher estimates exist [13]. The 5-year survival rate has been estimated to be below 3% [11].

For the other indications, our findings suggest the existence of long-term survival. For MBC, the observed median OS is lower compared to previously published estimates. A median survival of more than 30 months among women with MBC was observed in both single-centre and SEER population-based studies which assessed MBC survival outcomes [14, 15]. Both studies comprised younger patients comapared to this study. The single-centre study included 1,033 women with MBC diagnosed as early as 1990 and followed them up to 20 years [14] while the other study included 4,932 women with MBC diagnosed more recently (2010 – 2012) and a follow-up time of up to two years [15]. It appears that the potential underestimation may be due to patient selection in terms of e.g., age at diagnosis. The five and 10-year survival estimates, are more in line with other studies demonstrating long-term survival [16, 17]. For MCRC, our findings with regards to OS are in line with previous studies utilizing SEER data [18], and population-based Nordic registries [19]. The median OS was estimated to be between 11 and 13 months [18, 19]. Five-year survival was estimated to be approximately 10% [18, 19]. Similar estimates were reported for individuals with MMM. A Swedish study estimated a median relative survival of up to one year for MMM [20]. The study population consisted of all individuals with a reported advanced MM diagnosis in Sweden between 1990 and 2007 (*n* = 1,098). The follow-up time was up to 23 years.

Limited literature is available for long-term survival in ovarian cancer. SEER statistics show a survival rate of 27% at 5 years [21]. The 10-year survival has been estimated to be 8% [22].

For all indications, except for MNSCLC, the estimated cure fraction was below the observed 10-year survival. This indicates that the follow-up of this study may not suffice to demonstrate long-term survival. For MNSCLC the cure fraction was close to the estimated survival at 10 years. Considering the increased mortality for these individuals, the data may be considered sufficiently mature.

### Patient characteristics

Generally, age is considered a common prognostic factor for OS within oncology [11, 12, 15, 19–21]. In the Cox regression higher age was associated with worse prognosis. Potential reasons may be competing morbidity and mortality, fewer systemic or effective therapies offered, and decreased dose intensity [19]. A Swedish study found that increased age (> 70 years) was linked to fewer mammography screenings, fewer surgeries, and a decreased use of radiotherapy and chemotherapy among women with advanced BC [23]. This may be an indicator of decreased multidisciplinary management, compliance to clinical recommendations, and absence of clinical evidence for the treatment of older patients resulting in decreased OS [23]. Men with MNSCLC or MMM had a statistically significantly increased hazard of death compared to women. For MNSLCLC, this is in accordance with findings from previous research [11]. Reasons for a survival advantage among women with MNSCLC may include reproductive and hormonal factors [24], socioeconomic factors and difference in health-related behavior [25]. Potential survival differences across men and women with MMM may include behavioural differences resulting in e.g., diagnostic delay or biologic differences affecting the course of the disease [20, 26]. However, whether survival differs between men and women with MMM remains controversial [20, 26, 27]. Some studies observed that there is a female advantage in terms of survival, but it declines with more advanced stages and increasing tumour burden [26, 27]. With limited disease specific information, it is not possible to infer any causality. As population-based studies, compared to e.g., clinical trials, often include a relatively heterogenous MM population with different disease characteristics, a female advantage may become apparent despite selecting individuals with advanced disease only.

Distinct survival trends were observed regarding the year of diagnosis. Regardless of indication, individuals who were diagnosed more recently had a generally lower risk of dying compared to individuals who were diagnosed earlier. This observation has been made previously. Bar et al. reported an increasing 5-year survival among individuals with MNSCLC over time [12]. In fact, lung cancer has been linked to the most substantial increases in survival over time in relative terms [1]. Improvements in survival were also reported for the other indications. Potential reasons for a continuously improving survival may be effective novel treatments including immunotherapies, and clinical prognostic factors such as tumour biology [11, 14]. Additionally, it may be speculated that trends in the general population, such as an increasing life expectancy, translate into increased long-term survival among individuals with metastatic cancer. It should be noted that the survival data for individuals diagnosed between 2015 and 2018 is immature. However, considering conditional survival, the biggest opportunity to improve long-term survival may be during the initial years after diagnosis when treatment aims at disease control and potential cure [28]. Consequently, it is expected that the increased survival for those diagnosed more recently will eventually translate into increased long-term survival in the future. This emphasizes the need for more effective treatment options continuously facilitating improvements in OS and continuous monitoring of long-term survival to assess treatment advancements within oncology.

With increasing long-term survival, health care planning, economic, and organizational aspects become even more crucial to meet the needs of future patients and long-term survivors. The economic burden linked to cancer is substantial for public health systems, patients and their care givers [29]. As metastatic cancer becomes chronic and cancer treatments may take a toll on health, poorer, protracted health outcomes may be expected among long-term survivors. This in turn may translate into long-term economic burden [3]. Our study showed that long-term survival exists and potentially increases over time. These findings may be relevant for healthcare planning and resource allocation.

### Strengths and limitations

The limitations of this study are linked to the selection of individuals with metastatic malignancies. The estimated survival may not be fully representative for the respective indications. By combining the SCR and the SNPR using the PIN, both individuals diagnosed with de novo metastatic cancer or with a recurrent metastatic disease may have been identified. Valachis and colleagues aimed to identify individuals with MBC in national databases, with the aid of machine–learning by use of classifiers [30]. The authors included a total of 13,826 individuals with MBC between 2009 and 2016 where recurrent MBC was more prevalent (82% recurrent). According to the stated population size, the applied selection method in our study may underestimate the number of individuals, elly regarding recurrences. While this study only includes individuals with a registered diagnosis code indicating a secondary, distant malignancy, Valachis et al. also included individuals which did not have a relevant diagnosis code, but which were flagged as recurrent MBC by the classifiers [30]. Consequently, by selecting individuals based on registered diagnosis codes the number of individuals with any recurrent advanced malignancy may be underestimated or the secondary code may be unrelated to the primary cancer diagnosis. The same applies to individuals diagnosed with any de novo metastatic disease in this study. Selecting individuals based on the presence of a M1 code in combination with a relevant primary diagnosis may lead to an underestimation of the number of individuals due to unreported diagnosis codes.

Additionally, we only presented unadjusted OS which limits the comparability across indications as well as for calendar years and sex. Even though the presented Cox regression may corroborate the observed trends, the results should be interpreted with caution. A further limitation is the deficits in information on treatment and other, relevant prognostic factors. No causal relationships regarding long-term survival could be established. Further research is needed to determine patient and treatment characteristics of long-term survivors.

Regarding strengths, this is a unique study exploring the long-term survival of individuals with metastatic cancer. Generally, there is a need for data on long-term survival. By utilizing data from population-based registers, survival could be portrayed in an unselected population and real-world setting.

## Conclusion

This is the first population-based, longitudinal study looking at long-term survival in individuals with metastatic cancer, including de novo and recurrent disease. The use of Swedish national health registers made it possible to follow individuals over time and long-term survival was observed. However, more research is needed to fully assess factors impacting OS over time. Continuous monitoring of long-term survival is relevant for healthcare planning to meet the needs of future patients and potential long-term survivors.

## Supplementary Information

**Additional file 1: Figure 1. **Overall survival for all indications. 

**Additional file 2: Table 4. **Coxregression analysis, metastatic breast cancer.

**Additional file 3: Table 5. **Coxregression analysis, metastatic non-small cell lung cancer.

**Additional file 4: Table 6. **Coxregression analysis, metastatic colorectal cancer.

**Additional file 5: Table 7. **Coxregression analysis, metastatic ovarian cancer.

**Additional file 6: Table 8. **Coxregression analysis, metastatic malignant melanoma.

**Additional file 7: Table 9. **Overviewof best fitting mixture cure models for all indication.

## Data Availability

The datasets used and analysed during the current study are available from the corresponding author on reasonable request.
