# Supplementary material for: Overall survival of individuals with metastatic cancer in Sweden: a nationwide study
Source: BMC Public Health. 2022 Oct 14;22:1913. doi: 10.1186/s12889-022-14255-w (PMC9563107; doi:10.1186/s12889-022-14255-w)
Supplement: Supplementary file 1 — Additional file 1: Figure 1. Overall survival for all indications. [file 12889_2022_14255_MOESM1_ESM.docx]

Figure 1. Overall survival for all indications

1. *Metastatic breast cancer b) Metastatic non-small cell lung cancer*

*
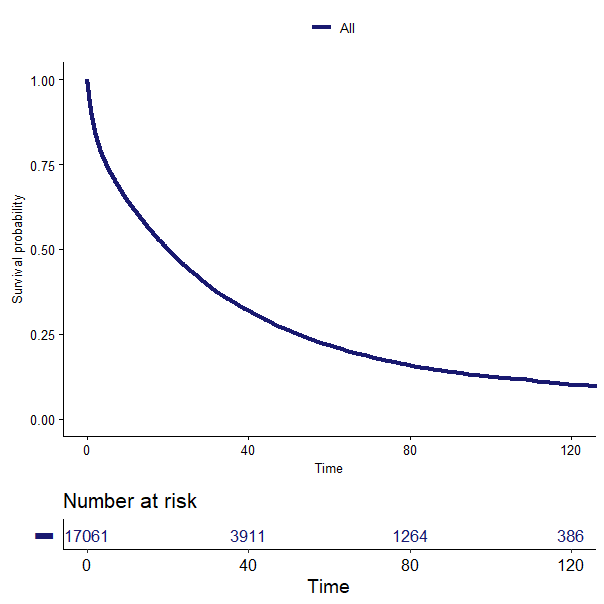

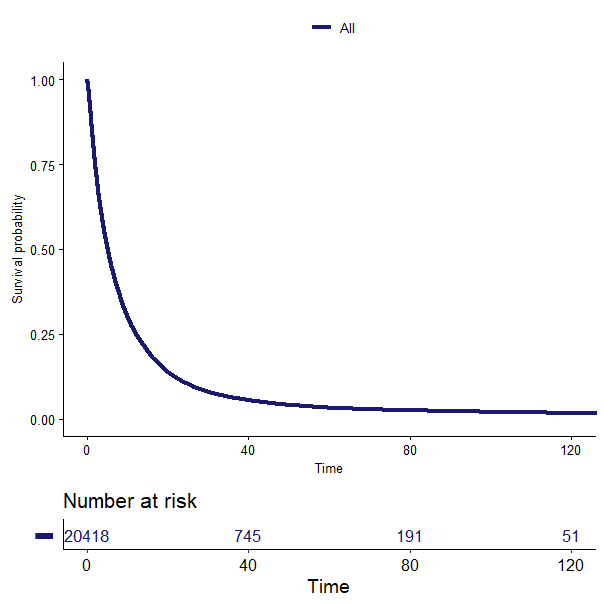
*

*c) Metastatic colorectal cancer d) Metastatic ovarian cancer*

*
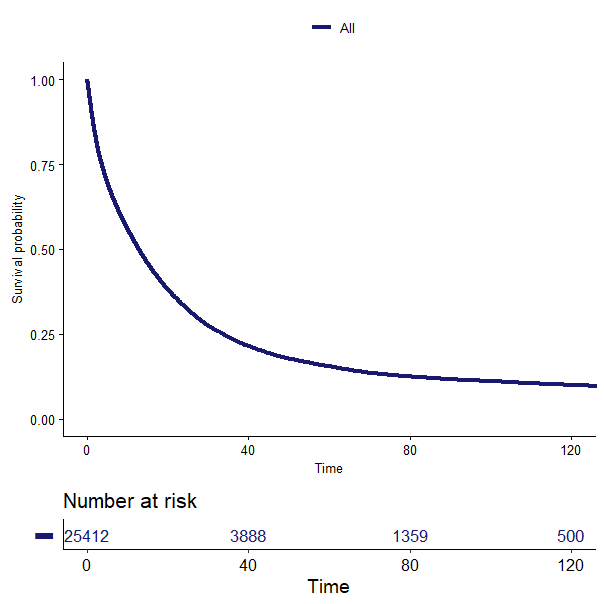

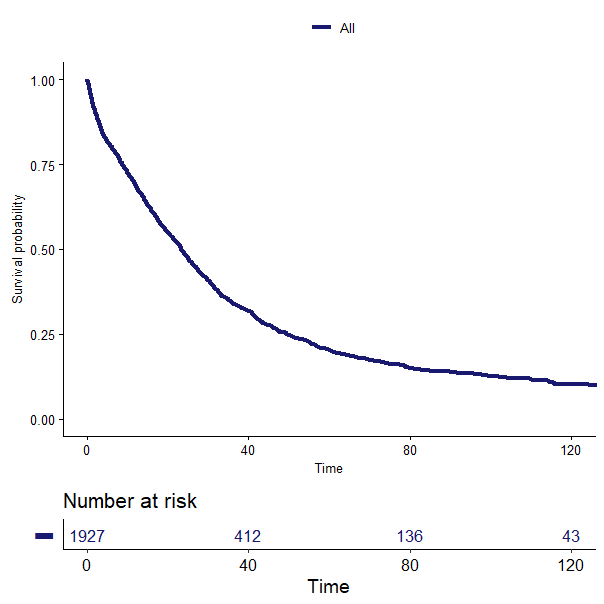
*

e) Metastatic malignant melanoma

*
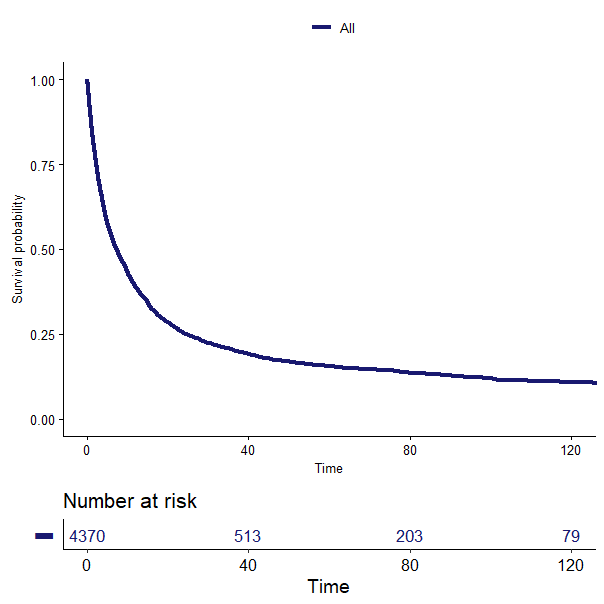
*
