# Supplementary material for: Overall survival of individuals with metastatic cancer in Sweden: a nationwide study
Source: BMC Public Health. 2022 Oct 14;22:1913. doi: 10.1186/s12889-022-14255-w (PMC9563107; doi:10.1186/s12889-022-14255-w)
Supplement: Supplementary file 2 — Additional file 2: Table 4. Coxregression analysis, metastatic breast cancer. [file 12889_2022_14255_MOESM2_ESM.docx]

Table 4. Cox regression analysis, metastatic breast cancer

| Variable | N | Crude hazard ratio | | Adjusted hazard ratio | |
| --- | --- | --- | --- | --- | --- |
|  |  | Hazard ratio (95% CI) | p | Hazard ratio (95% CI) | p |
| Diagnosis |  |  |  |  |  |
| MBC, *de novo* | 2,082 | 1.00 [Reference] |  | 1.00 [Reference] |  |
| MBC, recurrent | 14,979 | 1.01 (0.96 – 1.063) | 0.783 | 1.04 (0.98 – 1.10) | 0.164 |
| Age at diagnosis |  |  |  |  |  |
| <50 | 2,191 | 1.00 [Reference] |  | 1.00 [Reference] |  |
| 50-59 | 2,937 | 1.13 (1.05 – 1.21) | <0.001 | 1.12 (1.05 -1.20) | <0.001 |
| 60-69 | 4,241 | 1.25 (1.17 – 1.33) | <0.001 | 1.25 (1.17 – 1.33) | <0.001 |
| 70-79 | 4,030 | 1.47 (1.38 – 1.57) | <0.001 | 1.49 (1.40 – 1.59) | <0.001 |
| 80+ | 3,662 | 2.38 (2.24 – 2.53) | <0.001 | 2.40 (2.25 – 2.55) | <0.001 |
| Year of diagnosis |  |  |  |  |  |
| 2005-2009 | 6,063 | 1.00 [Reference] |  | 1.00 [Reference] |  |
| 2010-2014 | 6,118 | 0.88 (0.85 – 0.92) | <0.001 | 0.87 (0.84 – 0.91) | <0.001 |
| 2015-2018 | 4,880 | 0.74 (0.70 – 0.78) | <0.001 | 0.72 (0.69 – 0.76) | <0.001 |

CI: Confidence interval, MBC: Metastatic breast cancer
