# Supplementary material for: Overall survival of individuals with metastatic cancer in Sweden: a nationwide study
Source: BMC Public Health. 2022 Oct 14;22:1913. doi: 10.1186/s12889-022-14255-w (PMC9563107; doi:10.1186/s12889-022-14255-w)
Supplement: Supplementary file 3 — Additional file 3: Table 5. Coxregression analysis, metastatic non-small cell lung cancer. [file 12889_2022_14255_MOESM3_ESM.docx]

Table 5. Cox regression analysis, metastatic non-small cell lung cancer

| Variable | N | Crude hazard ratio | p | Adjusted hazard ratio | p |
| --- | --- | --- | --- | --- | --- |
|  |  | Hazard ratio (95% CI) |  | Hazard ratio (95% CI) |  |
| Diagnosis |  |  |  |  |  |
| MNSLC, *de novo* | 15,442 | 1.00 [Reference] |  | 1.00 [Reference] |  |
| MNSCLC, recurrent | 4,976 | 1.05 (1.02 – 1.09) | 0.003 | 1.05 (1.01 – 1.08) | 0.006 |
| Sex |  |  |  |  |  |
| Female | 9,650 | 1.00 [Reference] |  | 1.00 [Reference] |  |
| Male | 10,768 | 1.20 (1.17 – 1.24) | <0.001 | 1.19 (1.16 -1.22) | <0.001 |
| Age at diagnosis |  |  |  |  |  |
| <50 | 697 | 1.00 [Reference] |  | 1.00 [Reference] |  |
| 50-59 | 2,540 | 1.35 (1.23 – 1.48) | <0.001 | 1.35 (1.23 – 1.48) | <0.001 |
| 60-69 | 7,181 | 1.51 (1.39 – 1.65) | <0.001 | 1.52 (1.40 – 1.66) | <0.001 |
| 70-79 | 7,173 | 1.75 (1.60 – 1.90) | <0.001 | 1.78 (1.63 – 1.93) | <0.001 |
| 80+ | 2,827 | 2.15 (1.96 – 2.35) | <0.001 | 2.20 (2.01 – 2.41) | <0.001 |
| Year of diagnosis |  |  |  |  |  |
| 2005-2009 | 6,523 | 1.00 [Reference] |  | 1.00 [Reference] |  |
| 2010-2014 | 7,716 | 0.87 (0.84 – 0.90) | <0.001 | 0.86 (0.83 – 0.89) | <0.001 |
| 2015-2018 | 6,179 | 0.76 (0.73 – 0.79) | <0.001 | 0.74 (0.71 – 0.77) | <0.001 |

CI: Confidence interval, MNSCLC: metastatic non-small cell lung cancer
