# Supplementary material for: Overall survival of individuals with metastatic cancer in Sweden: a nationwide study
Source: BMC Public Health. 2022 Oct 14;22:1913. doi: 10.1186/s12889-022-14255-w (PMC9563107; doi:10.1186/s12889-022-14255-w)
Supplement: Supplementary file 4 — Additional file 4: Table 6. Coxregression analysis, metastatic colorectal cancer. [file 12889_2022_14255_MOESM4_ESM.docx]

Table 6. Cox regression analysis, metastatic colorectal cancer

| Variable | N | Crude hazard ratio | | Adjusted hazard ratio | |
| --- | --- | --- | --- | --- | --- |
|  |  | Hazard ratio (95% CI) | p | Hazard ratio (95% CI) | p |
| Diagnosis |  |  |  |  |  |
| MCRC, *de novo* | 12,401 | 1.00 [Reference] |  | 1.00 [Reference] |  |
| MCRC, recurrent | 13,011 | 0.89 (0.86 – 0.91) | <0.001 | 0.86 (0.84 – 0.88) | <0.001 |
| Sex |  |  |  |  |  |
| Female | 11,206 | 1.00 [Reference] |  | 1.00 [Reference] |  |
| Male | 14,206 | 0.94 (0.92 – 0.97) | <0.001 | 0.99 (0.96 – 1.01) | 0.355 |
| Age at diagnosis |  |  |  |  |  |
| <50 | 1,685 | 1.00 [Reference] |  | 1.00 [Reference] |  |
| 50-59 | 3,177 | 1.01 (0.94 – 1.09) | 0.744 | 1.01 (0.94 – 1.09) | 0.774 |
| 60-69 | 7,027 | 1.21 (1.14 – 1.29) | <0.001 | 1.22 (1.14 – 1.30) | <0.001 |
| 70-79 | 7,946 | 1.62 (1.52 – 1.72) | <0.001 | 1.64 (1.54 – 1.75) | <0.001 |
| 80+ | 5,577 | 2.79 (2.61 – 2.97) | <0.001 | 2.83 (2.65 – 3.02) | <0.001 |
| Year of diagnosis |  |  |  |  |  |
| 2005-2009 | 8,568 | 1.00 [Reference] |  | 1.00 [Reference] |  |
| 2010-2014 | 9,147 | 0.86 (0.83 – 0.88) | <0.001 | 0.84 (0.82 – 0.87) | <0.001 |
| 2015-2018 | 7,697 | 0.76 (0.73 – 0.79) | <0.001 | 0.74 (0.71 – 0.77) | <0.001 |

CI: Confidence interval, MCRC: metastatic colorectal cancer
