# Supplementary material for: Overall survival of individuals with metastatic cancer in Sweden: a nationwide study
Source: BMC Public Health. 2022 Oct 14;22:1913. doi: 10.1186/s12889-022-14255-w (PMC9563107; doi:10.1186/s12889-022-14255-w)
Supplement: Supplementary file 5 — Additional file 5: Table 7. Coxregression analysis, metastatic ovarian cancer. [file 12889_2022_14255_MOESM5_ESM.docx]

Table 7. Cox regression analysis, metastatic ovarian cancer

| Variable | N | Crude hazard ratio | | Adjusted hazard ratio | |
| --- | --- | --- | --- | --- | --- |
|  |  | Hazard ratio (95% CI) | p | Hazard ratio (95% CI) | p |
| Age at diagnosis |  |  |  |  |  |
| <50 | 195 | 1.00 [Reference] |  | 1.00 [Reference] |  |
| 50-59 | 381 | 1.12 (0.90 – 1.39) | 0.31 | 1.12 (0.90 – 1.40) | 0.315 |
| 60-69 | 652 | 1.35 (1.10 – 1.64) | 0.003 | 1.33 (1.09 – 1.60) | 0.005 |
| 70-79 | 518 | 1.58 (1.29 – 1.93) | <0.001 | 1.59 (1.29 – 1.90) | <0.001 |
| 80+ | 181 | 2.56 (2.02 – 3.26) | <0.001 | 2.55 (2.00 – 3.20) | <0.001 |
| Year of diagnosis |  |  |  |  |  |
| 2005-2009 | 584 | 1.00 [Reference] |  | 1.00 [Reference] |  |
| 2010-2014 | 755 | 0.97 (0.86 – 1.08) | 0.549 | 0.98 (0.87 – 1.10) | 0.72 |
| 2015-2018 | 588 | 0.68 (0.57 – 0.80) | <0.001 | 0.68 (0.58 – 0.80) | <0.001 |

CI: Confidence interval
