# Supplementary material for: Overall survival of individuals with metastatic cancer in Sweden: a nationwide study
Source: BMC Public Health. 2022 Oct 14;22:1913. doi: 10.1186/s12889-022-14255-w (PMC9563107; doi:10.1186/s12889-022-14255-w)
Supplement: Supplementary file 6 — Additional file 6: Table 8. Coxregression analysis, metastatic malignant melanoma. [file 12889_2022_14255_MOESM6_ESM.docx]

Table 8. Cox regression analysis, metastatic malignant melanoma

| Variable | N | Crude hazard ratio | | Adjusted hazard ratio | |
| --- | --- | --- | --- | --- | --- |
|  |  | Hazard ratio (95% CI) | p | Hazard ratio (95% CI) | p |
| Diagnosis |  |  |  |  |  |
| MMM, *de novo* | 235 | 1.00 [Reference] |  | 1.00 [Reference] |  |
| MMM, recurrent | 4,135 | 1.28 (1.01 – 1.49) | <0.01 | 1.33 (1.15 – 1.55) | <0.001 |
| Sex |  |  |  |  |  |
| Female | 1,737 | 1.00 [Reference] |  | 1.00 [Reference] |  |
| Male | 2.633 | 1.12 (1.05 – 1.20) | <0.01 | 1.14 (1.06 – 1.22) | <0.001 |
| Age at diagnosis |  |  |  |  |  |
| <50 | 623 | 1.00 [Reference] |  | 1.00 [Reference] |  |
| 50-59 | 639 | 1.06 (0.93 – 1.21) | 0.40 | 1.09 (0.95 – 1.24) | 0.209 |
| 60-69 | 1,043 | 1.17 (1.04 – 1.31) | 0.008 | 1.19 (1.06 – 1.33) | 0.004 |
| 70-79 | 1,139 | 1.48 (1.32 – 1.65) | <0.001 | 1.55 (1.38 – 1.74) | <0.001 |
| 80+ | 926 | 2.15 (1.91 – 2.41) | <0.001 | 2.24 (1.99 – 2.51) | <0.001 |
| Year of diagnosis |  |  |  |  |  |
| 2005-2009 | 1,343 | 1.00 [Reference] |  | 1.00 [Reference] |  |
| 2010-2014 | 1,574 | 0.91 (0.84 – 0.98) | 0.0119 | 0.88 (0.82 – 0.95) | 0.002 |
| 2015-2018 | 1,453 | 0.62 (0.57 – 0.68) | <0.001 | 0.59 (0.54 – 0.65) | <0.001 |

CI: Confidence interval, MMM: metastatic malignant melanoma
