# Supplementary material for: Overall survival of individuals with metastatic cancer in Sweden: a nationwide study
Source: BMC Public Health. 2022 Oct 14;22:1913. doi: 10.1186/s12889-022-14255-w (PMC9563107; doi:10.1186/s12889-022-14255-w)
Supplement: Supplementary file 7 — Additional file 7: Table 9. Overviewof best fitting mixture cure models for all indication. [file 12889_2022_14255_MOESM7_ESM.docx]

Table 9. Overview of best fitting mixture cure models for all indication

| Indication | Survival model | Parameter | Estimate | Lower CI (95%) | Upper CI (95%) | AIC | BIC |
| --- | --- | --- | --- | --- | --- | --- | --- |
| **Metastatic breast cancer** | Weibull | Cure fraction (%) | 4.00 | 3.10 | 5.10 | 114026.66 | 114049.90 |
|  |  | Shape | 0.72 | 0.71 | 0.73 |  |  |
|  |  | Scale | 29.18 | 28.04 | 30.36 |  |  |
| **Metastatic non-small cell lung cancer** | Generalized gamma | Cure fraction (%) | 1.50 | 1.30 | 1.80 | 120789.84 | 120821.53 |
|  |  | Location (mu) | 1.73 | 1.70 | 1.76 |  |  |
|  |  | Log-Scale (Sigma) | 1.26 | 1.25 | 1.28 |  |  |
|  |  | Shape (Q) | 0.27 | 0.23 | 0.31 |  |  |
| **Metastatic colorectal cancer** | Generalized gamma | Cure fraction (%) | 6.80 | 6.10 | 7.60 | 164883.69 | 164916.26 |
|  |  | Location (mu) | 2.58 | 2.55 | 2.62 |  |  |
|  |  | Log-Scale (Sigma) | 1.46 | 1.42 | 1.49 |  |  |
|  |  | Shape (Q) | 0.48 | 0.43 | 0.54 |  |  |
| **Metastatic ovarian cancer** | Generalized gamma | Cure fraction (%) | 8.60 | 6.50 | 11.30 | 12738.69 | 12760.95 |
|  |  | Location (mu) | 3.41 | 3.31 | 3.51 |  |  |
|  |  | Log-Scale (Sigma) | 1.04 | 0.94 | 1.15 |  |  |
|  |  | Shape (Q) | 1.12 | 0.91 | 1.33 |  |  |
| **Metastatic malignant melanoma** | Generalized gamma | Cure fraction (%) | 8.6 | 7 | 10.5 | 25474.28 | 25499.81 |
|  |  | Location (mu) | 1.85 | 1.77 | 1.94 |  |  |
|  |  | Log-Scale (Sigma) | 1.59 | 1.53 | 1.66 |  |  |
|  |  | Shape (Q) | 0.0951 | -0.0296 | 0.2198 |  |  |

AIC: Akaike information criterion, BIC: Bayesian information criterion, CI: Confidence interval
